# Resistance of hypervirulent *Klebsiella pneumoniae* to both intracellular and extracellular killing of neutrophils

**DOI:** 10.1371/journal.pone.0173638

**Published:** 2017-03-10

**Authors:** Lifeng Wang, Dingxia Shen, Hua Wu, Yanning Ma

**Affiliations:** Department of Microbiology, Chinese General Hospital of People’s Liberation Army, Beijing, China; Louisiana State University, UNITED STATES

## Abstract

Hypervirulent *Klebsiella pneumoniae* (HvKP) is hypermucoviscous organism, carrying genes of *rmpA* and *aerobactin*, causing serious community-acquired infection and metastatically spread in young healthy hosts. Neutrophils play an important role during innate immune response against bacterial infection by phagocytosis and neutrophil extracellular traps (NETs). Whether neutrophils can effectively defend against HvKP remains unclear. In this study, we observed that the HvKP was significantly more resistant to neutrophil-mediated phagocytosis and intracellular killing than classic *Klebsiella pneumoniae* (cKP) isolates. Although both HvKP and cKP induced NETs under scanning electron microscopy and confocal microscopy, more cKP than HvKP were trapped in NETs, and the killing by intracellular and extracellular mechanisms of neutrophils was detected only on cKP. Together, our results demonstrated that HvKP resisted to both intracellular and extracellular killing of neutrophils.

## Introduction

A new hypervirulent clinical variant of *Klebsiella pneumoniae* (HvKP) is emerging, and it has become the major pathogen associated with severe infections, such as pyogenic liver abscesses [1], pneumonia [2] and endophthalmitis [3]. It has been recognized that HvKP strains exhibit enhanced virulence features including producing more capsular polysaccharide, possessing anti-phagocytosis and causing distant metastases [4,5], and thus are much more invasive than classic *Klebsiella pneumoniae* (cKP). The majority of HvKP strains causing infections belong to serotype K1 or K2 [6]. HvKP can be identified by the string test and *rmpA* and *aerobactin* gene detection together [7].

Neutrophil-mediated response is essential for host to first combat bacterial infection. However, capsular polysaccharide (CPS) from *Klebsiella pneumoniae* (KP) increases resistance to neutrophil phagocytosis in vitro [8]. Previous studies have shown that serotype K1 of HvKP is significantly more resistant to neutrophil-mediated phagocytosis than non-K1 isolates [5]. Furthermore, in 2004 Brinkmann et al described a new mechanism of extracellular killing by neutrophils called NETosis [9]. Neutrophil extracellular traps (NETs) are fragile, extracellular, fiber-like structures composed of DNA, neutrophil antimicrobial factors and histone. Some bacteria have been reported to induce release of NETs [10] which can trap and kill a variety of microbes [11], such as *Staphylococcus aureus* [12], *Yersinia enterocolitica* and *Yersinia pseudotuberculosis* [13], *Escherichia coli* [14]. However, whether neutrophils release NETs in response to HvKP infection, and what the difference in formation of NETs between HvKP and cKP are currently unknown. In this study, we investigated the interaction of human neutrophils with HvKP and cKP in vitro.

## Materials and methods

### Bacterial strains

Fourty-five *Klebsiella pneumoniae* strains that were isolated from blood were investigated, including HvKP strains of serotype K1 (n = 16) and serotype K2 (n = 14), non-K1/K2 (cKP) isolates (n = 15). The HvKP-K1 and -K2 isolates were selected based on positive string test, gene amplification of *rmpA* and *aeobactin*. While cKP isolates were negative for string test and gene detection for *rmpA* and *aeobactin*. All forty-five strains were tested for phagocytosis and bactericidal activity assays. Only 6 strains (including 3 HvKP-K1 strains and 3 cKP strains) were tested for transmission electron microscopic observation and scanning electron microscopic observation. 18 strains (including 9 HvKP-K1 strains and 9 cKP strains) were tested for NETs immunofluorescence staining.

### Human neutrophils

Neutrophils were purified from freshly drawn blood of three healthy human volunteers who signed written consent prior to participation in the study. All experiments were approved by the Ethics Commettee of Chinese General Hospital of People’s Liberation Army. Polymorphprep™ (Axis-Shield) was used according to the manufacturer’s instructions to obtain neutrophil purity of approximately 95~97%. The major contaminant in neutrophil preparation was erythrocytes. Cells were counted in a Neubauer chamber. The purity was determined by the formula that the number of neutrophils divided by the number of total counted cells (at least 200 cells). The neutrophils were resuspended in phosphate-buffered saline (PBS, pH7.4) with concentration adjusted to 1 × 10^7^ cell/ml. The viability of the purified neutrophils was greater than 95% by trypan blue exclusion assay. Neutrophils were used immediately after isolation.

### Fluorescence labeling of bacteria

HvKP-K1, HvKP-K2 and cKP isolates incubated on Mueller-Hinton (MH) agar overnight at 37°C were adjusted to a concentration of 0.667 McFarland by spectrophotometer. The bacteria were placed in a 70°C water bath for 60 minutes, then 100 μl bacterial suspension was plated on MH agar to examine whether all bacteria were killed. The killed bacteria were washed with PBS and labeled with fluorescein isothiocyanate [FITC (0.1 mg/ml); Sigma Chemical Co.] in 0.1 M NaHCO_3_, pH 9.0 at 25°C for 1 hour. Unbound FITC was washed off with PBS for three times. FITC-labeled bacteria were resuspended to a concentration of 2 × 10^8^ cells/ml in PBS, aliquoted, and stored at -70°C. FITC-labeled KP strains (FITC-KP) were analyzed by flow cytometry (FACS Canto II, BD) to ensure the percentage of FITC-KP was greater than 99%.

### Phagocytosis assay

200 μl of FITC-KP suspension (representing 4 × 10^7^ cells) was added to a prewarmed mixture including 100 μl of a neutrophil suspension (representing 1 × 10^6^ cells), 100 μl of pooled normal human serum (10% v/v for opsonization), and 600 μl of PBS in a 10 × 75-mm Falcon™ polypropylene tube (BD, Franklin Lakes, NJ). The final volume was 1.0 ml and the multiplicity of infection (MOI, *Klebsiella pneumoniae*: neutrophils) was 40:1. Each tube was incubated at 37°C in a shaking water bath with continuous agitation except an unincubated tube served as control at 0-min. Tubes were transferred to an ice bath after incubation of 10, 30, 60 min. The superficial fluorescent was quenched by adding 100 μl Trypan Blue (0.04%) to determine the percent of neutrophils with ingested KP. The percentage of neutrophils ingested KP was assessed by flow cytometry (FACS canto II, BD). Neutrophil groups were gated according to forward scatter (FSC) and sideways scatter (SSC) combination. Then FITC positive cell groups were gated based on negative control (neutrophil suspension fixed with 2% paraformaldehyde and incubated with FITC-KP, then quenched), and positive control (neutrophils phagocytosed FITC-KP). A total of 10,000 neutrophils were processed. Phagocytic percentage was determined by the percentage of FITC positive neutrophils.

### Bactericidal activity assays

Neutrophils (1 × 10^6^ cells) were cultured to adhere to 24-well flat-bottom plates for 30 min. Then the prepared mixture (100 μl inactivated human normal serum, 200 μl bacterial suspension containing 4 × 10^7^ CFU and 600 μl PBS) was added to the 24-well plates with neutrophil adhered which served as an experimental test. A control test absent of neutrophils was set for every isolate. The plates were incubated at 37°C for 60 min. Then neutrophils were lysed with 0.1% TritonX 100 for 15 min on ice. Complete lysis of neutrophils was confirmed by microscopic observation. The suspension was diluted 1000-fold by the addition of PBS. Then 100 μl diluted suspension was plated on MH agar. Colony-forming units (CFUs) were enumerated the following day. Each experiment was repeated twice. The bacterial survival index was calculated with the equation: the number of CFUs in experimental test divided by the number of CFUs in control test. The results of every group indicated as mean ± standard deviations (S.D.). The survival index < 1.0 represented that there were bacteria killed by neutrophils. The survival index ≥ 1.0 represented that bacteria were not killed by neutrophils, or bacteria reproduced.

### Sample preparation for transmission electron microscopy (TEM)

Purified neutrophils were mixed with live HvKP-K1 or cKP, cultured for 30 min and 60 min as described above in phagocytosis assay. To get a visible cell cluster, we enlarged the final volume to 2.0 ml in TEM rather than 1.0 ml for phagocytosis assay, but the proportion of components and MOI were the same as phagocytosis assay. The phagocytosis reaction was stopped by placing the reaction tube to an ice bath. Neutrophils were collected by centrifugation (250 × *g*, 6 min) and were washed three times with cold PBS. The pellet was fixed in 3% (v/v) glutaraldehyde for 120 min, washed with 0.1 M PB for 10 min, three times in total. The cell pellet was dehydrated in a graded ethanol series and embedded in Eponate-12. Ultra-thin sections were stained with uranyl acetate and lead citrate and examined using transmission electron microscopy (HT7700; Japan). Approximately 100 microscopic fields were observed and 15 images were obtained from each isolate. The number of bacteria ingested by 20 neutrophils at 60 min was counted and the average bacterial number per neutrophil was then calculated for each isolate.

### Sample preparation for scanning electron microscopy (SEM)

Neutrophils (1 × 10^6^ cells) were cultured to adhere to coverslips treated with 0.01% polylysine (Sigma-Aldrich) in 24-well flat-bottom plates supplemented with 100 μl pooled normal human serum, treated with PMA (100 ng/ml) (Sigma-Aldrich) or challenged with 200 μl of HvKP-K1 or cKP suspension, containing 4 × 10^7^ CFU/ml each. The plates were incubated for 90 min in incubator with carbon dioxide. Next, neutrophils were fixed with 3% glutaraldehyde, then incubated with 1% osmium tetroxide and dehydrated with an ascending ethanol series. After dehydration and critical-point drying, the specimens were coated with gold and analyzed in a Hitachi S4800 scanning electron microscopy. The observation and the images obtained were the same as TEM. NET-bound CFUs were enumerated. Briefly, the number of bacteria trapped in NETs produced by 100 neutrophils was counted. Then the average number of bacteria for each neutrophil related NETs was calculated.

### Immunostaining

Neutrophils were challenged with HvKP-K1 or cKP for 90 min as described above in sample preparation for scanning electron microscopy. The neutrophils were fixed in 4% paraformaldehyde for 30 min at room temperature (RT), then washed with PBS for three times, after which the neutrophils were permeabilized in 0.2% Triton X-100 for 20 min at RT. Blocking was performed with 10% normal goat serum for 30 min at RT. Staining with primary antibodies [mouse anti-Myeloperoxidase antibody [2C7] (Abcam, ab25989) and rabbit anti-Histone H3 antibody (citrulline R2 + R8 + R17) (Abcam, ab5103)] was performed for 1 h at RT at 1:200 in 10% normal goat serum-PBS. Samples were washed with PBS for three times. Secondary antibody staining [goat anti-mouse IgG H&L-Alexa Fluor^®^ 647 (Abcam, ab150115) and goat anti-rabbit IgG H&L-Alexa Fluor^®^ 488 (Abcam, ab150077)] was performed for 30 min at RT at 1:500 in 10% normal goat serum-PBS. The unbound secondary antibodies were washed off using PBS for three times. Slides were mounted using mounting medium with DAPI (VECTASHIELD®, H-1200). Specimens were analyzed with a confocal microscopy (OLYMPUS FLUOVIEW1000). Approximately 100 microscopic fields were observed and 20 images obtained from each isolate.

### Statistical analysis

The differences of neutrophil phagocytosis and bacterial survival index among three groups were performed using one-way analysis of variance. Differences between groups were assessed by *t* test. All statistical tests were two sided. The p values less than 0.05 were considered to be statistically significant. Data were presented as mean ± standard deviation (S.D.).

## Results

### Phagocytosis of neutrophil against HvKP-K1, HvKP-K2 and cKP

**Fig 1:**
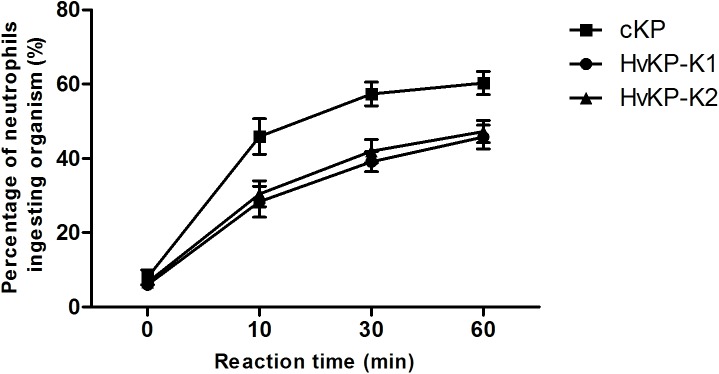
Phagocytosis of neutrophils against HvKP-K1, HvKP-K2 and cKP. The rate of phagocytosis against cKP (15 isolates) was higher than that against HvKP-K1 (16 isolates) or HvKP-K2 (14 isolates) at 10, 30, 60 min. The mean ± standard deviation (S.D.) of each group at each time point was calculated respectively. Statistics was performed using one-way analysis of variance for each time point. Differences between groups were assessed by *t* test. At 10, 30, 60 min, HvKP-K1 vs. cKP or HvKP-K2 vs. cKP: p < 0.05.

As shown in Fig 1, the rate of neutrophil phagocytosis against HvKP-K1, HvKP-K2 and cKP increased over time (for up to 60 min). The phagocytosis rate of HvKP-K1 and HvKP-K2 was similar. However, the phagocytosis rate of either HvKP-K1 or HvKP-K2 was lower than that of cKP at three time stages (10 min, 30 min and 60 min) (p < 0.05). The results indicated that HvKP-K1 and HvKP-K2 were more resistant to neutrophil phagocytosis compared with cKP.

### Bactericidal activity of human neutrophils

**Fig 2:**
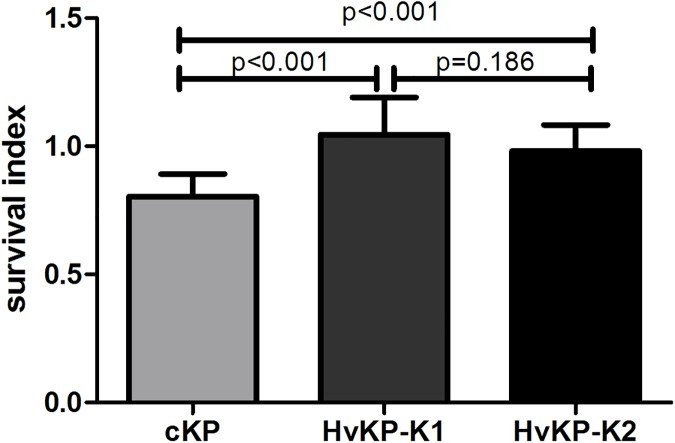
Survival of cKP, HvKP-K1 and HvKP-K2 within human neutrophils. The survival index was calculated with the equation described in the method. The survival index of cKP (15 isolates) was lower than that of HvKP-K1 (16 isolates) or HvKP-K2 (14 isolates). Each strain was repeated twice and averaged. Then the mean ± standard deviation (S.D.) of each group was calculated. Statistics was performed using one-way analysis of variance. Differences between groups were assessed by *t* test. HvKP-K1 vs. cKP or HvKP-K2 vs. cKP: p < 0.001.

The survival index of HvKP-K1 and HvKP-K2 within neutrophils was 1.0450 ± 0.1455 and 0.9820 ± 0.1013, respectively. There was no significant difference between them. For cKP, the survival index was 0.8038 ± 0.0876, and it was significantly lower than that of HvKP-K1 and HvKP-K2 (Fig 2).

### Observation under transmission electron microscopy

**Fig 3:**
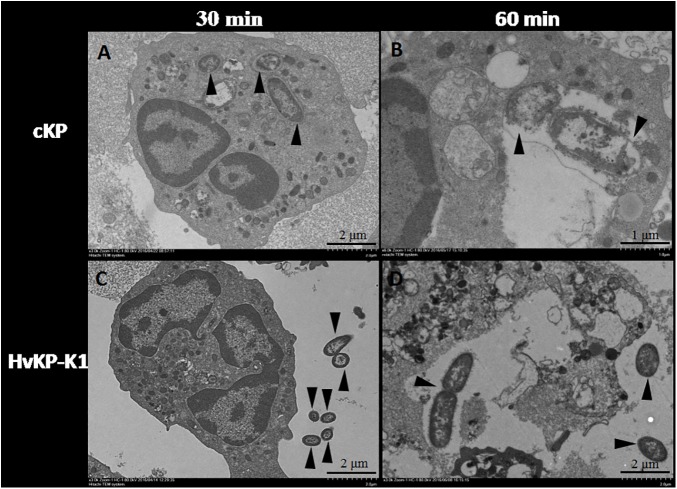
Examination under transmission electron microscopy. Phagocytosed cKP (3 isolates, A) and HvKP-K1 (3 isolates, C) by neutrophils at 30 min (×3K); lysed cKP lack of intact cell wall (3 isolates, B) and binary fission multiplying HvKP-K1 (3 isolates, D) at 60min (×6K). Bacteria were indicated by black arrows.

Neutrophils were infected with HvKP-K1 or cKP for 30 min. As shown in Fig 3A and 3C, neutrophils were able to ingest more cKP than HvKP-K1, with no morphological changes of phagocytosed HvKP-K1 and cKP at this time point. When neutrophils were infected with HvKP-K1 or cKP for 60 min, the number of cKP and HvKP-K1 ingested per neutrophil was 2.17 ± 0.24 and 1.33 ± 0.23. The bacterial cells of cKP ingested by neutrophils displayed incomplete cell surface and seemed to be lysed at this time point (Fig 3B), while the bacterial cell wall of HvKP-K1 ingested by neutrophils remained intact, and the cell division of HvKP-K1 was visible. Interestingly, some neutrophils ingested HvKP-K1 started to break (Fig 3D).

### Observation of NETs induced by HvKP-K1 and cKP by scanning electron microscopy and confocal microscopy

**Fig 4:**
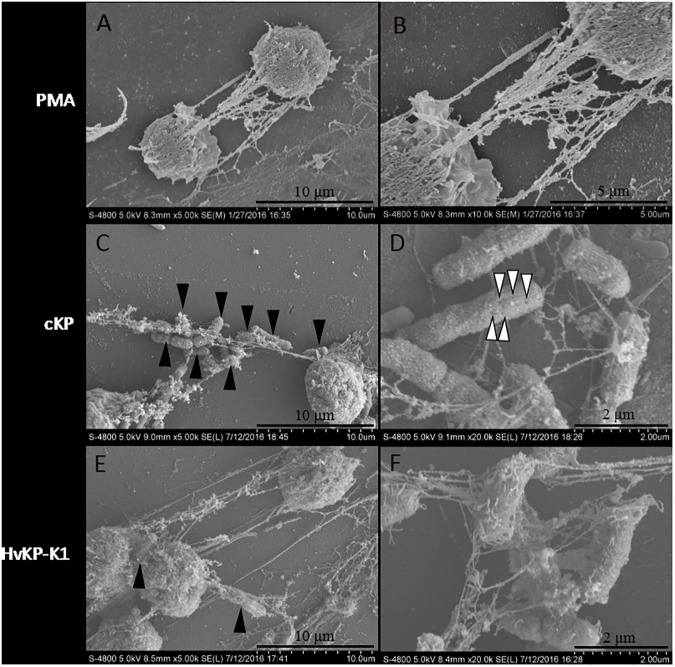
NETs under scanning electron microscopy. NETs induced by PMA (A and B), cKP (3 isolates, C and D), and HvKP-K1 (3 isolates, E and F). More cKP (C) than HvKP-K1 (E) were trapped in NETs by magnification of 5K. The pores (indicated by white arrows) on the surface of cKP, but not on the surface of HvKP-K1 were observed by magnification of 20K (D and F). Bacteria were indicated by black arrows.

In cultures of neutrophils with PMA for 90 min, neutrophils could produce NETs (Fig 4A and 4B), which was also observed in the co-cultures of neutrophils with either HvKP-K1 or cKP. Although both HvKP-K1 (Fig 4E and 4F) and cKP (Fig 4C and 4D) were able to induce neutrophils to release fiber-like NETs, composed of smooth interweaved-fiber structures and globular domains, the amount of bacteria trapped in NETs was different. The average number of cKP and HvKP-K1 for each neutrophil related NETs was 19.27 ± 5.13 and 2.30 ± 0.52, respectively. The results confirmed that NETs trapped a lot more cKP (Fig 4C) than HvKP-K1 (Fig 4E). Moreover, the trapped cKP showed pores on the surface, but this phenomenon didn’t appear on trapped HvKP-K1 (Fig 4D and 4F).

**Fig 5:**
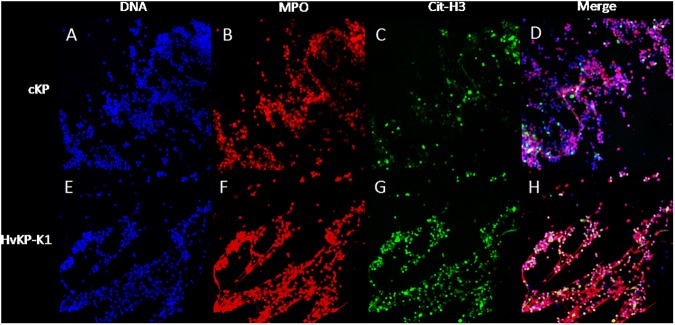
Immunofluorescence staining under confocal microscopy. Neutrophils challenged by cKP (9 isolates, A-D) and HvKP-K1 (9 isolates, E-H) were stained for DNA (DAPI, blue, A and E), myeloperoxidase (MPO, red, B and F) and citrullinated histone H3 (cit-H3, green, C and G). The merged images of cKP (D) and HvKP-K1 (H) illustrated the characteristic neutrophil extracellular traps. Original magnification 40×.

DNA (Fig 5A and 5E), myeloperoxidase (MPO, Fig 5B and 5F) and histone H3 (Fig 5C and 5G) were selected for staining of NETs. Immunofluorescence analysis confirmed that the visualized structures were classic characteristics of NETs in both cKP group (Fig 5D, merge) and HvKP-K1 group (Fig 5H, merge).

## Discussion

Neutrophils are essential effector cells of the innate immune system that are extremely important for the first-line defense against bacterial infections. Phagocytosis and NETs are the intracellular and extracellular antimicrobial mechanisms for neutrophils, respectively. How these mechanisms work in clearing HvKP inspired our effort to this study.

In this study, we demonstrated that: (i) HvKP was resistant to phagocytosis and intracellular killing of neutrophils; (ii) Both HvKP-K1 and cKP could induce NETs release, but HvKP was resistant to trapping and killing by NETs.

Phagocytosis assay showed the rate of phagocytosis for HvKP was lower than that for cKP. Furthermore, the observation that neutrophils ingested less HvKP-K1 than cKP under transmission electron microscopy confirmed the results of phagocytosis. Therefore, resistance to phagocytosis was an indicator of virulence for HvKP.

Following phagocytosis, microbes are exposed to reactive oxygen species and antimicrobial peptides that effectively kill and digest most microorganisms [15]. The results of bactericidal activity assay indicated that HvKP was resistant to neutrophil intracellular killing. In contrast, the survival index of cKP was lower than that of HvKP, and more than that, the phagocytosed organisms of cKP begun to lyse at 60 min under transmission electron microscopy, which clearly indicated intracellular killing within neutrophils.

Interestingly, multiplying organisms of HvKP-K1 by two-division were observed under transmission electron microscopy, which provided further evidence that HvKP-K1 possessed the ability to resist intracellular killing. Lin’s study showed that the HvKP-K1 causing liver abscesses resisted to neutrophil-mediated intracellular killing which contributed to the dissemination and establishment of distant metastases [5].

In addition, it had been noticed that the neutrophils phagocytosed HvKP-K1 started to lyse without similar finding for the neutrophils phagocytosed cKP. Kobayashi and his colleagues found that there were two fundamental outcomes after the interaction of bacteria with neutrophils. On one hand, bacteria induced an apoptosis differentiation program in human neutrophils and contributed to resolution of bacterial infection, on the other hand, microorganisms such as *Streptococcus*. *pyogenes* accelerated the apoptosis program of neutrophils, resulting in pathogen survival and diseases [16]. Therefore, HvKP-K1 causing neutrophil lysis revealed its hypervirulence and lead to severe infection in clinical. How HvKP-K1 realized this function and whether bacterial components including capsular polysaccharides worked remained to be investigated.

NETs contained neutrophil antimicrobial factors, including neutrophil elastase and myeloperoxidase [9], and played an important role in trapping extracellular killing [9,17,18]. Wartha et al indicated that capsules of *Streptococcus pneumoniae* reduced trapping and protected bacteria from killing by NETs [19]. Our results demonstrated that both HvKP-K1 and cKP isolates were able to induce the release of NETs by human neutrophils in vitro (Fig 4), and the immunostaining showed that DNA, histone H3 and MPO could be detected within interweaved-fiber structures, confirmed NETs formation. However, under scanning electron microscopy, more cKP were trapped by NETs than HvKP-K1, and the pores on the surface of cKP (not on the surface of HvKP) were found. Our enumeration under scanning electron microscopy is semi-quantitative. In 2010 Berends et al used NET entrapment assays for quantitative analysis of bacterial entrapment by activated neutrophils [20]. Therefore, we speculated that HvKP strains resisted extracellular killing of neutrophils. Those observations indicated that HvKP-K1 possessed ability to resist trapping and killing by NETs compared with cKP.

In summary, our results demonstrated that HvKP was not only resistant to phagocytosis and intracellular killing by neutrophils, but also resistant to trapping and killing of NETs produced by neutrophils. More research is required to understand the mechanisms and to identify the important virulence components from HvKP for potential vaccine and therapeutic targets.
